# Ultrasound-based radiomics nomogram combined with clinical features for the prediction of central lymph node metastasis in papillary thyroid carcinoma patients with Hashimoto’s thyroiditis

**DOI:** 10.3389/fendo.2022.993564

**Published:** 2022-08-19

**Authors:** Peile Jin, Jifan Chen, Yiping Dong, Chengyue Zhang, Yajun Chen, Cong Zhang, Fuqiang Qiu, Chao Zhang, Pintong Huang

**Affiliations:** ^1^ Department of Ultrasound in Medicine, Zhejiang University School of Medicine Second Affiliated Hospital, Zhejiang University, Hangzhou, China; ^2^ Research Center of Ultrasound in Medicine and Biomedical Engineering, Zhejiang University School of Medicine Second Affiliated Hospital, Zhejiang University, Hangzhou, China; ^3^ Research Center for Life Science and Human Health, Binjiang Institute of Zhejiang University, Hangzhou, China

**Keywords:** ultrasound, central lymph node metastasis, Hashimoto’s thyroiditis, radiomics, papillary thyroid carcinoma

## Abstract

**Background:**

Hashimoto thyroiditis (HT) is the most common autoimmune thyroid disease and is considered an independent risk factor for papillary thyroid carcinoma (PTC), with a higher incidence of PTC in patients with HT.

**Objective:**

To build an integrated nomogram using clinical information and ultrasound-based radiomics features in patients with papillary thyroid carcinoma (PTC) with Hashimoto thyroiditis (HT) to predict central lymph node metastasis (CLNM).

**Methods:**

In total, 235 patients with PTC with HT were enrolled in this study, including 101 with CLNM and 134 without CLNM. They were divided randomly into training and validation datasets with a 7:3 ratio for developing and evaluating clinical features plus conventional ultrasound features (Clin-CUS) model and clinical features plus radiomics scores (Clin-RS) model, respectively. In the Clin-RS model, the Pyradiomics package (V1.3.0) was used to extract radiomics variables, and LASSO regression was used to select features and construct radiomics scores (RS). The Clin-CUS and Clin-RS nomogram models were built using logistic regression analysis.

**Results:**

Twenty-seven CLNM-associated radiomics features were selected using univariate analysis and LASSO regression from 1488 radiomics features and were calculated to construct the RS. The integrated model (Clin-RS) had better diagnostic performance than the Clin-CUS model for differentiating CLNM in the training dataset (AUC: 0.845 vs. 0.778) and the validation dataset (AUC: 0.808 vs. 0.751), respectively.

**Conclusion:**

Our findings suggest that applying an ultrasound-based radiomics approach can effectively predict CLNM in patients with PTC with HT. By incorporating clinical information and RS, the Clin-RS model can achieve a high diagnostic performance in diagnosing CLNM in patients with PTC with HT.

## Introduction

Hashimoto thyroiditis (HT) is the most common autoimmune thyroid disease and is characterized by increased thyroid volume, parenchymal lymphocyte infiltration, hypothyroidism, and elevated serum-related specific antibodies. HT is considered an independent risk factor for papillary thyroid carcinoma (PTC), with a higher incidence in patients with HT (1).

Patients with HT tend to develop reactive central cervical lymph node hyperplasias. Moreover, the sonographic features of these nodes were round, sharp, heterogeneous echogenicity, and the absence of fatty hilum, similar to metastatic cervical lymph nodes in patients with PTC (2, 3). Furthermore, the sensitivity of conventional ultrasound for metastatic central cervical nodes in patients with PTC was less than 50% (3). Thus, if PTC is complicated with HT, it may increase the difficulty of preoperative identification of central lymph node metastasis (CLNM) (4).

However, the presence or absence of CLNM directly affects the surgical method (5). Several studies have examined CLNM incidence in patients with PTC and HT. They found that CLNM incidence was significantly lower in patients with PTC with HT than in patients with PTC only (6–8). However, in clinical practice, patients with PTC with HT who underwent surgery had more resected lymph nodes than those without HT (7), which led to a greater rate of complications, including recurrent laryngeal nerve injury and hypoparathyroidism (9–11). Ahn et al. also reported that patients with PTC and thyroiditis were more likely to have undergone a central compartment neck dissection at the time of thyroidectomy than those without thyroiditis (8). Therefore, it is necessary to improve CLNM evaluation in patients with PTC with HT.

Some studies have focused on the association between the ultrasonic characteristics of PTC and CLNM. Li et al. demonstrated that conventional ultrasound and shear wave elastography of PTC could predict CLNM with the area under the curve (AUCs) of 0.749 and 0.774, respectively (12). Tao et al. also found that CEUS in PTC was associated with CLNM, with an AUC of 0.727 (13). However, the limited ultrasonic features recognized by the naked eye may restrict the diagnostic capability of ultrasound imaging. Moreover, the interpretation of ultrasonic features is operator- and experience-dependent, leading to further inter-reader variation (14).

Radiomics is a rapidly developing method that utilizes many image texture and morphology features and provides more quantitative and objective information for the diagnosis and prognostic analysis of diseases (15, 16). The integration of radiomics and clinical information may further increase diagnostic performance.

Therefore, this study aimed to explore the associations of a radiomics model with CLNM in patients with PTC with HT and propose a nomogram model integrated with ultrasound-based radiomics and clinical information for predicting CLNM in this population.

## Materials and methods

### Patient enrollment

**Figure 1:**
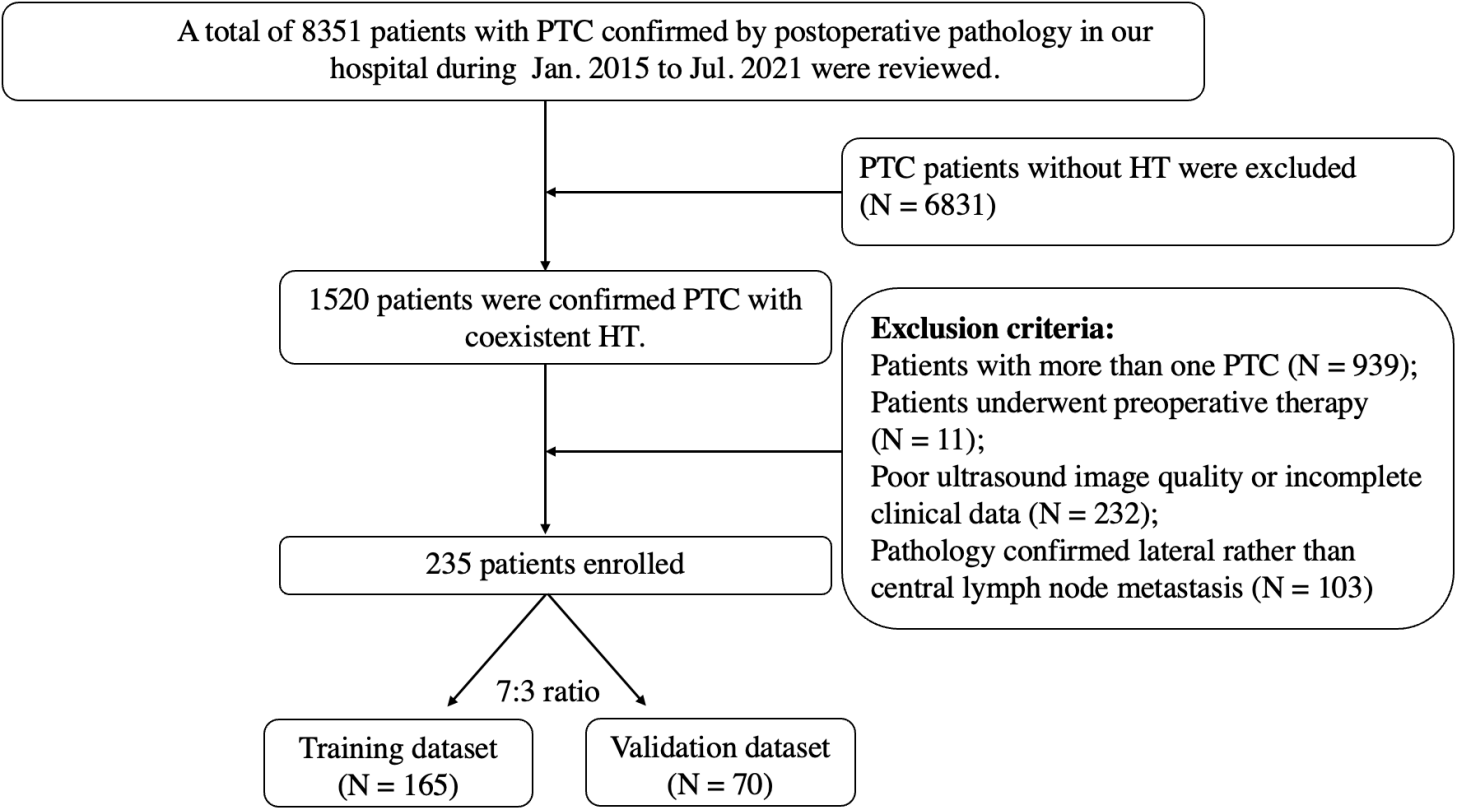
Flowchart of patient selection for differentiating CLNM of PTC patients with HT. PTC, papillary thyroid carcinoma; HT, Hashimoto’s thyroiditis; CLNM, central lymph node metastasis.

From January 2015 to July 2021, 235 patients with pathological results of PTC and HT were enrolled, including 101 with CLNM and 134 without CLNM. All patients underwent conventional ultrasound (CUS), fine-needle aspiration biopsy with Bethesda V-VI and hematological examination preoperatively and underwent either lobectomy or total thyroidectomy. Cervical lymph node dissection was routinely performed (**Figure 1**).

### Inclusive and Exclusive Criteria

#### Inclusive criteria

Patients were enrolled based on the following criteria:1) patients with unifocal PTC. 2) Pathological examination confirming PTC with HT after surgery. 3) Patients with clear B-mode ultrasound imaging of the thyroid. 4) Patients with complete clinical information such as clinical features (age, gender, tumor dimension, and tumor location), CUS features (such as echogenicity, aspect ratio, boundary, margin), and thyroid function (such as TT3, FT3, TT4, FT4, TSH).

#### Exclusive criteria

1) Patients with multifocal PTCs. 2) Poor US imaging quality. 3) Patients who had undergone preoperative therapy, such as radiofrequency and microwave ablation, were excluded.

### Conventional US and color doppler US

US examination of patients with PTC was performed by experienced radiologists. The ultrasound machine included Resona7 (Mindray, Shenzhen, China), ESAOTE (MyLAb 90 X-vision, Italy), Aplio 500 (Toshiba Medical Systems, Tokyo, Japan), and Logic E9 (GE Healthcare, Wauwatosa, USA). All selected thyroid nodules were evaluated for the following US features echogenicity (marked hypoechoic, hypoechoic, and iso/hyperechoic), aspect ratio (>1 or ≤1), boundary (clear and unclear), margin (well-defined and ill-defined), calcification (no calcification, macrocalcification, and microcalcification), and blood flow in the nodule (avascularity, peripheral vascularity, limited vascularity, and strip-like vascularity).

### Region of interest segmentation and feature extraction

First, the patients were randomly divided into training and validation sets in a 7:3 ratio. Second, the picture file format was changed to jpg and imported into the PyCharm (Community 2021.1) software. The region of interest (ROI) was manually segmented by an experienced radiologist and confirmed by another. The ROI included two parts (thyroid nodules and thyroid glands). All nodules were confirmed as PTC by pathological biopsy. This process was accomplished with the help of the Labelme (3.16.7) software package. Finally, the radiomics data were extracted using the python package pyradiomics (V1.3.0) (17), and 1488 radiomics features from the thyroid (744 from thyroid nodules and 744 from thyroid glands) were extracted in this study.

### Feature selection and radiomics model establishment

**Figure 2:**
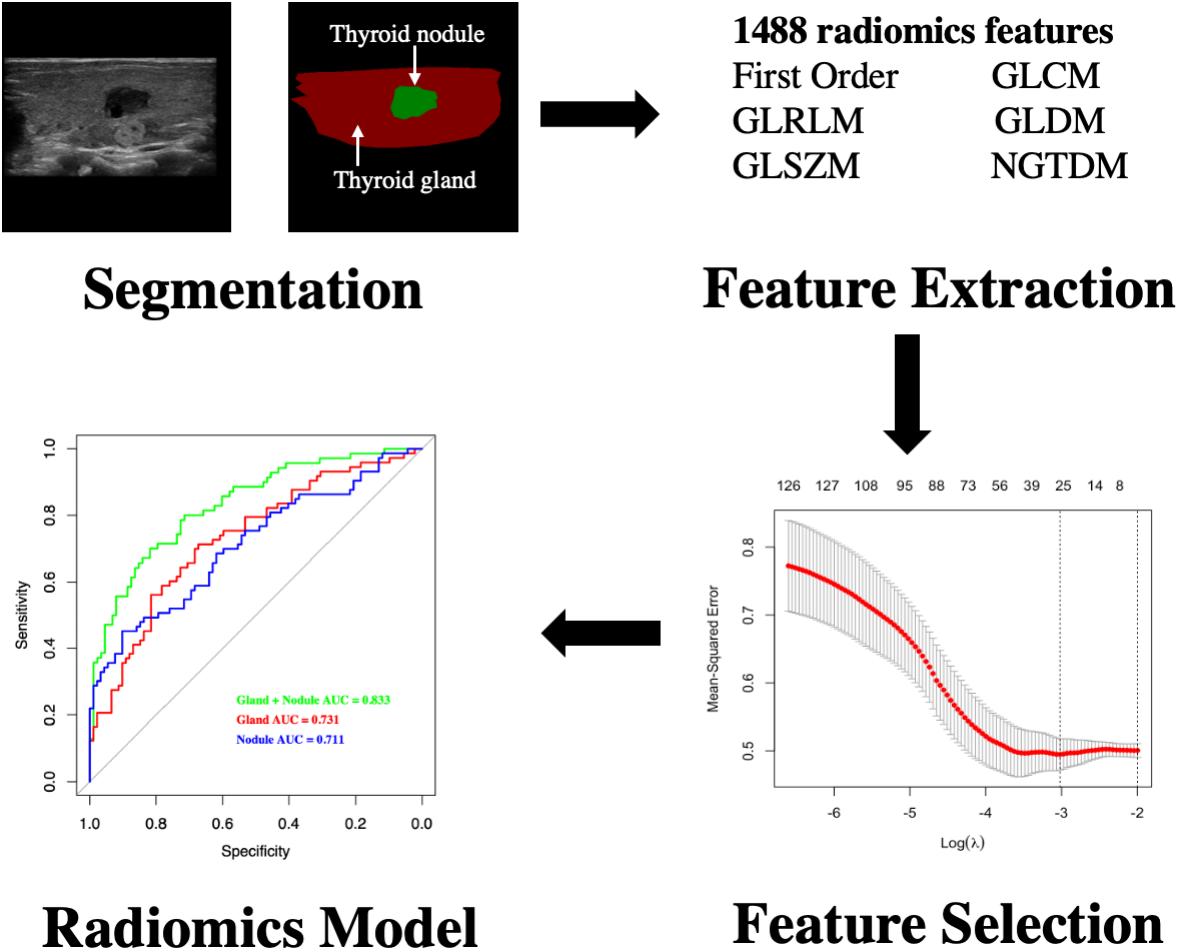
Flowchart of development of radiomics model for differentiating CLNM of PTC patients with HT. CLNM, central lymph node metastasis; PTC, papillary thyroid carcinoma; HT, Hashimoto’s thyroiditis.

The thyroid was divided into two parts: the thyroid gland and the thyroid nodule. We also established the Gland, Nodule, and Gland + Nodule models based on radiomics features extracted from the thyroid nodules and thyroid gland (**Figure 2**).

Independent *t*-test and least absolute shrinkage and selection operator (LASSO) regression were used to select significant features (18). Next, we calculated the radiomics scores (RS) based on regression technology. Receiver operating characteristic (ROC) curves were used to compare the diagnostic performance (**Figure 2**).

### Statistical analysis

Continuous data with a normal distribution are shown as mean ± standard deviation, and data with a non-normal distribution are shown as medians (quartile intervals). Student’s t-test and Mann-Whitney U test were used for comparisons between the two groups. Categorical data were expressed as numbers (percentages) and compared using the Mann-Whitney U test or chi-squared test. The distributions of our data were measured using the Shapiro-Wilk test. All statistical analyses were performed using SPSS24 (SPSS/IBM, Chicago, IL, USA), R (version 4.1.1) software, and RStudio (Version 1.4.1717).

Significant clinical features, CUS features, and RS of the training dataset in the univariate analysis were included in the multiple logistic regression model. Multiple logistic regression analysis with forward selection was used to identify the independent risk factors for predicting CLNM in the training dataset. Odds ratios (ORs) with relative 95% confidence intervals (CIs) were calculated to determine the relevance of all the potential predictors of CLNM. Considering the potential influence of clinical features and CUS features for each patient, different nomogram models were built using logistic regression, including the RS-Clin (radiomics scores + clinical features) and Clin-CUS (clinical features + conventional ultrasound features) models. The diagnostic performance for discriminating patients with PTC and CLNM from patients with PTC without CLNM was determined by the area under the receiver operating curve (AUC-ROC). Delong’s test was used to compare AUCs. A P-value < 0.05 indicated a statistically significant difference for all statistical methods.

## Results

### Baseline characteristics of patients with PTC with HT

In total, 101 (43.0%) and 134 (57.0%) patients with CLNM and without CLNM were enrolled, respectively. The clinical characteristics of the patients are shown in **Table 1**. Patients with CLNM were younger than those without CLNM (35 (30-44.5) vs. 44 (34.8-57), P<0.001).

**Table 1:** Baseline clinical data of all PTC patients with HT.

Variables	CLNM		P-Value
	Yes (N = 101)	No (N = 134)	
**Clinical features**			
Age	35 (30-44.5)	44 (34.8-57)	<0.001
Age < 41 y	71 (70.3%)	57 (42.5%)	<0.001
Age ≥ 41 y	30 (29.7%)	77 (57.5%)	
Gender			0.038
Male	13 (12.9%)	7 (5.2%)	
Female	88 (87.1%)	127 (94.8%)	
Tumor Dimension	0.8 (0.6-1.4)	0.7 (0.5-0.8)	<0.001
≥ 0.9cm	50 (49.5%)	27 (20.1%)	<0.001
< 0.9cm	51 (50.5%)	107 (79.9%)	
Location			0.708
Left	48 (47.5%)	62 (46.3%)	
Right	49 (48.5%)	69 (51.5%)	
Isthmus	4 (4.0%)	3 (2.2%)	
**CUS features**			
Echogenicity			0.133
iso/hyperechoic	3 (3.0%)	1 (0.7%)	
hypoechoic	71 (70.3%)	84 (62.7%)	
marked hypoechoic	27 (26.7%)	49 (36.6%)	
Aspect ratio			0.519
≤1	38 (37.6%)	56 (41.8%)	
>1	63 (62.4%)	78 (58.2%)	
Boundary			0.843
clear	42 (41.6%)	54 (40.3%)	
unclear	59 (58.4%)	80 (59.7%)	
Margin			<0.001
well-defined	33 (32.7%)	74 (55.2%)	
ill-defined	68 (67.3%)	60 (44.8%2)	
Calcification			<0.001
NO	29 (28.7%)	75 (56.0%)	
macrocalcification	11 (10.9%)	16 (11.9%)	
microcalcification	61 (60.4%)	43 (32.1%)	
Blood flow			0.089
avascularity	59 (58.4%)	90 (67.2%)	
peripheral vascularity	3 (3.0%)	7 (5.2%)	
limited vascularity	36 (35.6%)	37 (27.6%)	
strip-like vascularity	3 (3.0%)	0 (0%)	
**Thyroid function**			
TT3	1.6 ± 0.3	1.6 ± 0.2	0.352
FT3	4.4 (4.0-4.7)	4.4 ± 0.5	0.486
TT4	97.4 ± 21.2	99.1 (83.2-112.3)	0.642
FT4	12.8 (12.1-13.9)	12.9 ± 1.5	0.762
TSH	1.6 (1.1-2.1)	1.8 (1.1-2.6)	0.089
TG	2.5 (0.4-16.8)	4.0 (0.7-14.0)	0.656
TGAb	67.1 (23.0-261.2)	65.9 (19.6-214.1)	0.729
TPOAb	21.3 (1.1-404.6)	42.0 (3.7-263.9)	0.615
**Radiomics Feature**			
RS	0.13 ± 0.73	-0.51 ± 0.52	<0.001

Continuous data with normal distribution were shown as mean ± standard deviation and data with a non-normal distribution were shown as median (quartile interval).

PTC, papillary thyroid carcinoma; CLNM, central lymph node metastasis; HT, Hashimoto's thyroiditis; CUS, conventional ultrasound; TT3, total triiodothyronine; FT3, free triiodothyronine; TT4, total thyroxine; FT4, free thyroxine; TSH, thyroid stimulating hormone; Tg, thyroid globulin; TGAb, anti-thyroglobulin antibodies; TPOAb, thyroidperoxidase antibodies; RS, radiomics scores.

Of the patients with CLNM, 12.9% were male, and 5.2% were male in patients without CLNM (P=0.038).

We also found a statistically significant difference in tumor dimension (P < 0.001), margin (P < 0.01), calcification (P < 0.001), and RS (P < 0.001) between patients with and without CLNM.

No statistical difference was found in the blood index of thyroid function (TT3, P = 0.352; FT3, P = 0.486; TT4, P = 0.642; FT4, P = 0.762; TSH, P = 0.089; TG, P = 0.656; TGAb, P = 0.729; TPOAb, P = 0.615).

### Univariate analysis of clinical features, CUS features, and RS for CLNM

In this study, the patients were randomly divided into training and validation datasets in a 7:3 ratio. There were almost no significant differences in the other variables (all P > 0.05), except for TGAb (P = 0.030) between the two groups (**Table 2**). The training dataset found that age, gender, tumor diameter, and RS were significantly different between CLNM-positive and CLNM-negative patients (P < 0.05, **Supplemental Table 1**). The same findings were also seen in the validation dataset, whereas gender was not (P = 0.661) (**Supplemental Table 2**). Concerning CUS features, ill-defined margins and microcalcifications were significantly different between the two groups in the training dataset (P < 0.05). However, only the ill-defined margin in the validation dataset was statistically different between the two populations (P = 0.040).

**Table 2:** Baseline clinical data in the training and validation datasets.

Variable	Training dataset	Validation dataset	P-value
	N = 165	N = 70	
**Clinical features**			
Age	40 (33 – 53.5)	40.44 ± 12.06	0.209
Age < 41 y	87 (52.7%)	41 (58.6%)	0.411
Age ≥ 41 y	78 (47.8%)	29 (41.4%)	
Gender			
Male	15 (9.1%)	5 (7.1%)	0.625
Female	150 (90.9%)	65 (92.9%)	
Tumor Diameter	0.8 (0.5 – 1.0)	0.7 (0.5 – 1.0)	
Diameter ≥ 0.9cm	55 (33.3%)	22 (31.4%)	0.776
Diameter < 0.9cm	110 (66.7%)	48 (68.6%)	
Location(L/R/I)			
Left	75 (45.5%)	35 (50.0%)	0.8593
Right	85 (51.5%)	33 (47.1%)	
Isthmus	5 (3.0%)	2 (2.9%)	
**CUS features**			
Echogenicity			
iso/hyperechoic	3 (1.8%)	1 (1.4%)	0.854
hypoechoic	107 (64.8%)	48 (68.6%)	
marked hypoechoic	55 (33.3%)	21 (30.0%)	
Aspect ratio			
≤1	67 (40.6%)	27 (38.6%)	0.771
>1	98 (59.4%)	43 (61.4%)	
Boundary			
clear	69 (41.8%)	27 (38.6%)	0.643
unclear	96 (58.2%)	43 (61.4%)	
Margin			
well-defined	79 (47.9%)	28 (40.0%)	0.267
ill-defined	86 (52.1%)	42 (60.0%)	
Calcification			
NO	70 (42.4%)	32 (45.7%)	0.889
macrocalcification	19 (11.5%)	8 (11.4%)	
microcalcification	76 (46.1%)	30 (42.9%)	
Blood flow			
avascularity	105 (63.6%)	44 (62.9%)	0.871
peripheral vascularity	6 (3.6%)	4 (5.7%)	
limited vascularity	52 (31.5%)	21 (30.0%)	
strip-like vascularity	2 (1.2%)	1 (1.4%)	
**Thyroid function**			
TT3	1.56 ± 0.22	1.62 (1.43 – 1.72)	0.456
FT3	4.38 ± 0.51	4.38 (4.15 – 4.79)	0.271
TT4	97.10 ± 20.82	98.13 ± 22.12	0.735
FT4	12.96 ± 1.48	12.99 ± 1.77	0.906
TSH	1.67 (1.10 – 2.41)	1.70 (1.23 – 2.29)	0.831
TG	3.03 (0.47 – 12.75)	3.22 (0.75 – 19.47)	0.264
TGAb	83.31 (29.02 – 231.79)	41.76 (10.54 – 214.81)	0.030
TPOAb	31.94 (2.11 – 287.14)	52.93 (3.04 – 403.72)	0.265
**Radiomics features**			
RS	-0.28 (-0.60 – 0.11)	-0.15 ± 0.65	0.386

PTC, papillary thyroid carcinoma; CLNM, central lymph node metastasis; HT, Hashimoto's thyroiditis; CUS, conventional ultrasound; TT3, total triiodothyronine; FT3, free triiodothyronine; TT4, total thyroxine; FT4, free thyroxine; TSH, thyroid stimulating hormone; TG, thyroid globulin; TGAb, anti-thyroglobulin antibodies; TPOAb, thyroid peroxidase antibodies; RS, radiomics scores.

### Performance of three radiomics models in PTC patients with HT

**Figure 3:**
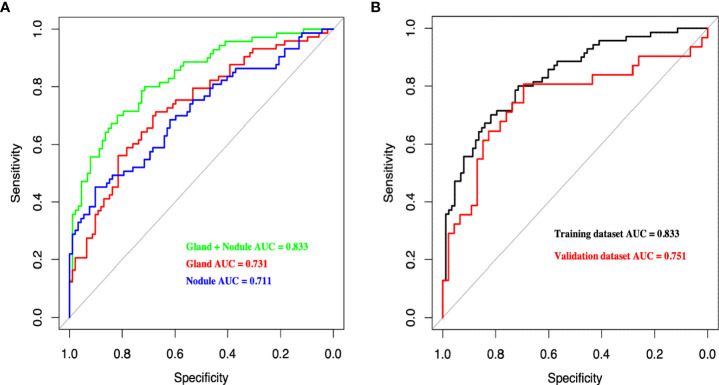
**(A)** Receiver operating characteristic curves of different radiomics models for predicting CLNM in the training dataset. **(B)** Receiver operating characteristic curves of Gland + Nodule model in training and validation dataset. CLNM, central lymph node metastasis.

In total, 1488 radiomics variables from the thyroid (744 from thyroid nodules and 744 from the thyroid gland) were extracted in this study, and three models (Nodule, Gland, and Gland + Nodule models) were constructed. The AUC of the Gland + Nodule model was better than that of the Nodule (0.833 vs. 0.731, P = 0.033) and Gland (0.833 vs. 0.711, P = 0.022) models (**Figure 3**). In the Gland + Nodule model, the LASSO procedure was performed, and 27 radiomics features were selected, including one from GLCM, five from GLRLM, four from GLSZM, four from the first order, five from NGTDM, and eight from wavelet (**Table 3**). Moreover, the AUCs of the Gland + Nodule model were 0.833 and 0.751 for the training and validation datasets, respectively (**Figure 3**). According to the 27 radiomics features, we calculated RS in the training dataset, and RS showed a significant difference between the two populations (P < 0.001, **Supplemental Table 1**).

**Table 3:** The Class of Extracted variables.

Variables Class	Thyroid nodule	Thyroid gland
GLCM	1	0
GLRLM	4	1
GLSZM	3	1
First order	2	2
NGTDM	2	3
Wavelet	5	3

GLCM, Grey-Level Co-occurrence Matrix; GLRLM, Gray-Level Run-Length Matrix; GLSZM, Gray-level size zone matrix; NGTDM, Neighborhood Gray Tone Difference Matrix.

### Multivariate analysis of clinical features, CUS features, and RS for CLNM

After multivariate analysis, four variables were selected: RS, male, age < 41 years, and tumor diameter ≥ 0.9 cm. The results showed that RS was the best predictor for CLNM, with an OR of 5.55, and it was almost one and a half times that of Male (OR = 3.81) and two times that of age < 41 years (OR = 2.83). We also considered tumor diameter ≥ 0.9 cm (OR = 2.10) because previous studies (19–22) reported that a larger tumor diameter was an independent predictor for CLNM (**Table 4**). However, CUS features (ill-defined margins and microcalcifications) were not independent signatures for predicting CLNM (**Supplemental Table 3**).

**Table 4:** Multivariable analysis of clinical features and RS for predicting in PTC patients with HT in the training dataset.

Parameter	OR	95% CI	P-value
Age < 41 y	2.83	1.34 - 6.21	0.008
Tumor diameter ≥0.9 cm	2.10	0.91 - 4.88	0.079
Male	3.81	1.07 - 15.68	0.046
RS	5.55	2.61 - 13.46	<0.001

OR, odds ratios; CI, confidence intervals; RS, radiomics scores.

### Diagnostic performance of clinical features, CUS features, and RS for CLNM

**Figure 4:**
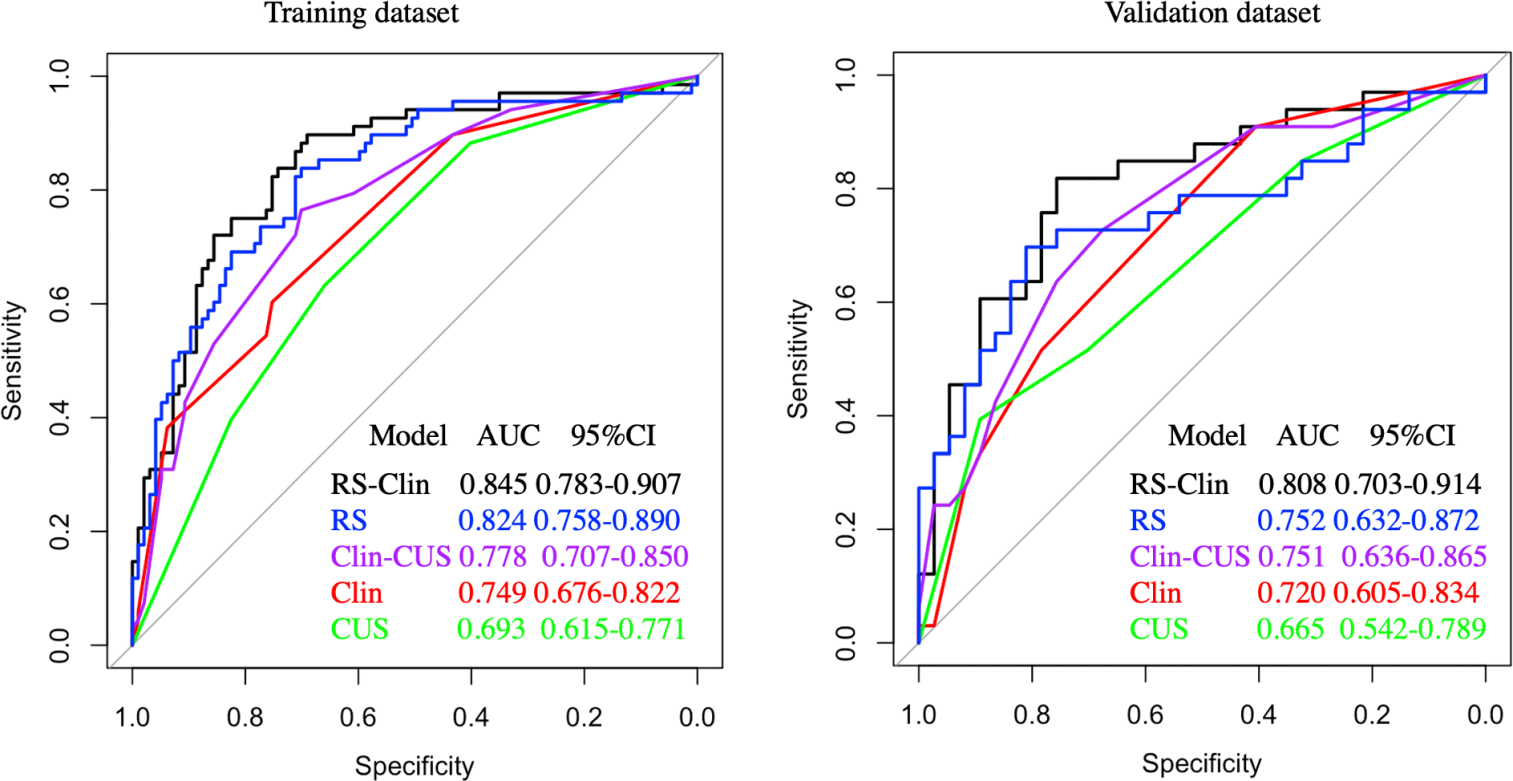
Receiver operating characteristic curves of different predictive models for predicting CLNM in training and validation dataset. CLNM, central lymph node metastasis; Clin-RS, clinical data + radiomics scores; RS, radiomics scores; Clin-CUS, clinical data + conventional ultrasound; Clin, clinical data; CUS, conventional ultrasound.

Based on clinical features (age, gender, and tumor diameter), CUS features (margin and calcification), and RS, we constructed Clin, CUS, RS, Clin-CUS, and RS-Clin models. The RS-Clin model yielded the best performance, with an AUC of 0.845 and 0.808 in the training and validation datasets, respectively. Compared to the RS-Clin model, the AUCs of the Clin, CUS, and Clin-CUS models were 0.749, 0.693, and 0.778, respectively, in the training dataset (P = 0.001, P <0.001, and P = 0.019, respectively) (**Figure 4**). Furthermore, the AUC of the RS model was 0.824, which was not statistically different from that of the RS-Clin model (P = 0.298). In the validation dataset, the AUCs of the Clin, CUS and Clin-CUS models were 0.72, 0.665, 0.751, and 0.752, respectively (**Figure 4**).

### Nomogram construction and validation

**Figure 5:**
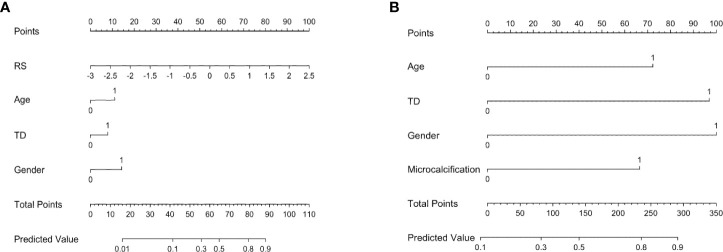
Nomogram for the RS-Clin **(A)** and Clin-CUS **(B)** model for predicting the probability of CLNM. RS, radiomics scores; Age 0: age ≥ 41y, 1: age < 41y; TD, tumor diameter 0: TD < 0.9cm, 1: TD ≥ 0.9cm; Gender 0: Female, 1: Male; Microcalcification 0: No/macrocalcification, 1: microcalcification; RS-Clin, radiomics scores + Clinical features; Clin-CUS, Clinical features + CUS features.

After multivariate regression analysis, we constructed two prediction models, the RS-Clin (radiomics scores + clinical features) model and Clin-CUS (clinical features + conventional ultrasound features) model. The nomogram graphics of the RS-Clin and Clin-CUS models are shown in **Figure 5**.

**Figure 6:**
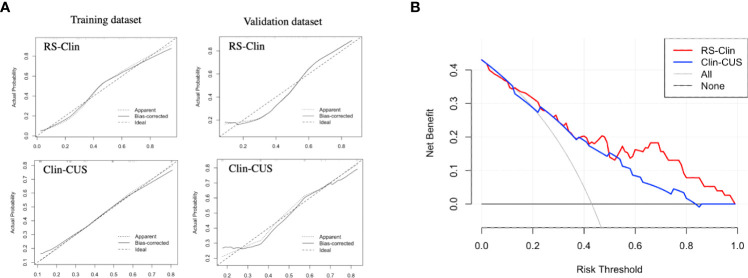
**(A)** Calibration curve of RS-Clin and Clin-CUS model for predicting the probability of CLNM in the training dataset and validation dataset. **(B)** Decision curve analysis of two nomogram models for predicting the probability of CLNM in the validation dataset. RS-Clin, radiomics scores + Clinical features; Clin-CUS, Clinical features + CUS features; CLNM, central lymph node metastasis.

The calibration and decision curves of the RS-Clin and Clin-CUS models corresponded well between the prediction results and observations in the validation dataset (**Figure 6**).

## Discussion

HT plays a vague role in patients with PTC and is reported to be a risk factor for PTC (23, 24) and a protective factor in terms of PTC prognosis (24, 25). Central lymph node metastasis is one the most important risk factors for postoperative local recurrence of thyroid cancer. Prophylactic central compartment neck dissection could improve disease-free survival in patients with PTC (26). Patients with PTC with HT have a better prognosis (8, 22, 24, 25, 27), but they appear to undergo more extensive central compartment neck dissection and are more likely to have a greater number of lymph nodes resected than patients with PTC only (6, 7). However, it is even more difficult to distinguish patients with the potential risk of CLNM from patients with PTC with HT since no accurate diagnostic blood indexes have been reported, and thyroid ultrasound imaging is compromised. In addition, many signs that determine the prognosis of the disease are related to various invisible factors to the human eye. Currently, most predictive variables rely on a single examination, such as ultrasound features, thyroid function examination, and some clinical risk factors. The rapid development of radiomics may provide a solution for the difficulty in CLNM diagnosis, which can analyze images quantitatively, mine the image information deeply, and find features that the naked eye cannot detect. Therefore, building an integrated model based on clinical indices and radiomics models in patients with PTC with HT is of great significance in clinical practice.

This study used a radiomics approach to extract radiomics features from two-dimensional ultrasonic thyroid images of patients with PTC with HT. Three radiomics models were constructed (Gland, Nodule, and Gland + Nodule models). The AUCs of the three models were 0.833, 0.731, and 0.711, respectively, which demonstrated that both the thyroid gland and thyroid nodules contributed to the identification of CLNM. This result was supported by Tong et al., who reported that the radiomics features of thyroid nodules can predict CLNM in patients with PTC (28). However, only a small number of patients with HT were included in this study. In addition, we found that radiomics features from the thyroid gland were valuable and contributed to predicting the status of CLNM in patients with PTC.

Therefore, we calculated the RS based on the Gland + Nodule model. Then, through univariate and multivariate analyses, we found that age, gender, tumor dimension, margin, calcification, and RS were significantly different between the two groups, which was similar to previous reports (19–21, 29, 30). Based on the clinical features, CUS features, and RS, we constructed the Clin, CUS, RS, Clin-CUS, and RS-Clin models. Among these models, the RS-Clin model yielded the best performance, with AUC of 0.845 and 0.808 in the training and validation datasets, respectively. In the training dataset, the AUCs of the RS-Clin model were statistically different from those of the Clin, CUS, and Clin-CUS models, but there was no difference compared to the RS model, which means that RS was the best predictive factor for identifying the status of CLNM in patients with PTC and HT. Furthermore, we established nomograms based on the RS-Clin and Clin-CUS models for clinical decision-making.

This study had several limitations. First, this retrospective study may have led to a potential selection bias. Second, this was a single-center study, and a multicenter study with a larger number of patients is required. Third, this study focused on patients with unifocal PTC, while the features of multifocal PTCs were a risk factor for CLNM, and further study is necessary. Fourth, we did not compare the US features of central lymph nodes with and without metastasis. Fifth, all patients included in this study were diagnosed with Bethesda V-VI by FNA before surgery, but given the false negative of fine needle aspiration, some patients with thyroid cancer were not included, which will cause selection bias; therefore, a prospective study is necessary.

## Conclusion

Our findings demonstrate that the application of the radiomics approach to ultrasound images could effectively predict CLNM in patients with PTC with HT. By incorporating clinical information and RS, the RS-Clin model can achieve a high diagnostic performance in the diagnosis of CLNM in patients with PTC with HT.

## Data availability statement

The original contributions presented in the study are included in the article/**Supplementary Material**. Further inquiries can be directed to the corresponding author.

## Ethics statement

The studies involving human participants were reviewed and approved by Zhejiang University School of Medicine Second Affiliated Hospital. The ethics committee waived the requirement of written informed consent for participation.

## Author contributions

PJ, JC and YD contributed equally to this study. PJ and JC were responsible for the conception and design of this study. PJ and YD contributed to data analysis and writing of the manuscript. CYZ, YC and CoZ contributed to data collection. FQ and ChZ contributed to data analysis and manuscript preparation. JC and PH contributed to writing-reviewing. All authors contributed to the article and approved the submitted version.

## Funding

The study was supported by the National Key R&D Program of China [grant number 2018YFC0115900] and Natural Science Foundation of Zhejiang Province [grant number LY16H180005, LQ19H180004, LQ20H180009, LQ21H180007].

## Conflict of interest

The authors declare that the research was conducted in the absence of any commercial or financial relationships that could be construed as a potential conflict of interest.

## Publisher’s note

All claims expressed in this article are solely those of the authors and do not necessarily represent those of their affiliated organizations, or those of the publisher, the editors and the reviewers. Any product that may be evaluated in this article, or claim that may be made by its manufacturer, is not guaranteed or endorsed by the publisher.
