# Supplementary material for: Ultrasound-based radiomics nomogram combined with clinical features for the prediction of central lymph node metastasis in papillary thyroid carcinoma patients with Hashimoto’s thyroiditis
Source: Front Endocrinol (Lausanne). 2022 Aug 19;13:993564. doi: 10.3389/fendo.2022.993564 (PMC9439618; doi:10.3389/fendo.2022.993564)
Supplement: Supplementary file 1 [file DataSheet_1.docx]

**Supplemental Information**

| **Supplemental Table 1. Univariate analysis of clinical features, CUS features, and RS for predicting CLNM in the training dataset** | | | | | |
| --- | --- | --- | --- | --- | --- |
| **Variable** | **CLNM** | | | | **P-value** |
|  | Yes (N=68) | | No (N=97) | |  |
| **Clinical features** |  | |  | |  |
| Age | | 35.5 (32-47) | 44 (35-57) | | 0.002 |
| Age < 41 y | 47 | | 40 | | <0.001 |
| Age ≥ 41 y | 21 | | 57 | |  |
| Sex |  | |  | | 0.036 |
| Male | 10 | | 5 | |  |
| Female | 58 | | 92 | |  |
| Tumor Diameter | 0.9 (0.6-1.3) | | 0.7 (0.5-0.8) | | 0.001 |
| Diameter ≥ 0.85cm | 35 | | 20 | | <0.001 |
| Diameter < 0.85cm | 33 | | 77 | |  |
| Location(L/R/I) |  | |  | | 0.942 |
| Left | 32 | | 43 | |  |
| Right | 34 | | 51 | |  |
| Isthmus | 2 | | 3 | |  |
| **CUS features** |  | |  | |  |
| Echogenicity |  | |  | | 0.344 |
| iso/hyperechoic | 2 | | 1 | |  |
| hypoechoic | 47 | | 60 | |  |
| marked hypoechoic | 19 | | 36 | |  |
| Aspect ratio |  | |  | |  |
| ≤1 | 24 | | 43 | | 0.245 |
| >1 | 44 | | 54 | |  |
| Boundary |  | |  | |  |
| clear | 29 | | 40 | | 0.857 |
| unclear | 39 | | 57 | |  |
| Margin |  | |  | | 0.007 |
| well-defined | 24 | | 55 | |  |
| ill-defined | 44 | | 42 | |  |
| Calcification |  | |  | | <0.001 |
| NO | 17 | | 55 | |  |
| macrocalcification | 8 | | 11 | |  |
| microcalcification | 43 | | 33 | |  |
| Blood flow |  | |  | | 0.192 |
| avascularity | 39 | | 66 | |  |
| peripheral vascularity | | 2 | 4 | |  |
| limited vascularity | 25 | | 27 | |  |
| strip-like vascularity | 2 | | 0 | |  |
| **Thyroid function** |  | |  | |  |
| TT3 | 1.54 ± 0.23 | | | 1.57 ± 0.21 | 0.375 |
| FT3 | 4.34 ± 0.57 | | | 4.41± 0.47 | 0.368 |
| TT4 | 97.08 ± 21.67 | | | 97.11 ± 20.33 | 0.992 |
| FT4 | 13.01 ± 1.39 | | | 12.94 ± 1.54 | 0.768 |
| TSH | 1.57 (1.07 - 2.19) | | | 1.72 (1.13 - 2.64) | 0.169 |
| TG | 1.55 (0.39 - 13.22) | | | 3.75 (0.8 - 12.4) | 0.309 |
| TgAb | 83.23 (30.78 - 227.38) | | | 83.31 (28.31 - 233.52) | 0.949 |
| TPOAb | 17.41 (1.35 - 334.76) | | | 37.75 (3.09 - 246.15) | 0.782 |
| **Radiomics features** | |  |  | |  |
| RS | 0.13 ± 0.74 | | -0.55 ± 0.54 | | <0.001 |
| PTC: papillary thyroid carcinoma; CLNM: central lymph node metastasis; HT: Hashimoto's thyroiditis; CUS: conventional ultrasound; TT3: total triiodothyronine; FT3: free triiodothyronine; TT4: total thyroxine; FT4: free thyroxine; TSH: thyroid stimulating hormone; TG: thyroid globulin; TGAb: anti-thyroglobulin antibodies; TPOAb: thyroidperoxidase antibodies; RS: radiomics scores | | | | | |

| **Supplemental Table 2. Univariate analysis of clinical features, CUS features and RS for predicting CLNM in the validation dataset** | | | |
| --- | --- | --- | --- |
| **Variable** | **CLNM** | | **P-value** |
|  | Yes (N=33) | No (N=37) |  |
| **Clinical features** |  |  |  |
| Age | 36.4 ± 10.2 | 44.1 ± 12.6 | 0.007 |
| Age < 41 y | 24 (72.7%) | 17 (45.9%) | 0.023 |
| Age ≥ 41 y | 9 (27.3%) | 20 (54.1%) |  |
| Gender |  |  | 0.661 |
| Male | 3 (9.1%) | 2 (5.4%) |  |
| Female | 30 (90.9%) | 35 (94.6%) |  |
| Tumor Diameter | 0.8 (0.7-1.5) | 0.6 (0.5-0.8) | 0.009 |
| Diameter ≥ 0.9cm | 15 (45.5%) | 7 (18.9%) | 0.017 |
| Diameter < 0.9cm | 18 (54.5%) | 30 (81.1%) |  |
| Location(L/R/I) |  |  | 0.420 |
| Left | 16 (48.5%) | 19 (51.4%) |  |
| Right | 15 (45.5%) | 18 (48.6%) |  |
| Isthmus | 2 (6.1%) | 0 (0%) |  |
| **CUS features** |  |  |  |
| Echogenicity |  |  | 0.434 |
| iso/hyperechoic | 1 (3.0%) | 0 (0%) |  |
| hypoechoic | 24 (72.7%) | 24 (64.9%) |  |
| marked hypoechoic | 8 (24.2%) | 13 (35.1%) |  |
| Aspect ratio |  |  |  |
| ≤1 | 14 (42.4%) | 13 (35.1%) | 0.532 |
| >1 | 19 (57.6%) | 24 (64.9%) |  |
| Boundary |  |  |  |
| clear | 13 (39.4%) | 14 (37.8%) | 0.894 |
| unclear | 20 (60.6%) | 23 (62.2%) |  |
| Margin |  |  | 0.040 |
| well-defined | 9 (27.3%) | 19 (51.4%) |  |
| ill-defined | 24 (72.7%) | 18 (48.6%) |  |
| Calcification |  |  | 0.072 |
| NO | 12 (36.4%) | 22 (59.5%) |  |
| macrocalcification | 3 (9.1%) | 5 (13.5%) |  |
| microcalcification | 18 (54.5%) | 10 (27%) |  |
| Blood flow |  |  | 0.669 |
| avascularity | 20 (60.6%) | 24 (64.9%) |  |
| peripheral vascularity | 1 (3.0%) | 3 (8.1%) |  |
| limited vascularity | 11 (33.3%) | 10 (27.0%) |  |
| strip-like vascularity | 1 (3.0%) | 0 (0%) |  |
| **Thyroid function** |  |  |  |
| TT3 | 1.57 (1.41 - 1.65) | 1.62 ± 0.04 | 0.163 |
| FT3 | 4.39 (4.09 - 4.78) | 4.45 ± 0.07 | 0.902 |
| TT4 | 97.94 ± 25.74 | 98.29 ± 18.67 | 0.948 |
| FT4 | 12.75 (11.99 - 14.03) | 12.77 ± 0.23 | 0.596 |
| TSH | 1.64 (1.23 - 2.08) | 1.98 ± 0.18 | 20.312 |
| TG | 2.78 (1.56 - 24.28) | 5.79 (0.74 - 16.47) | 0.514 |
| TgAb | 36.83 (10.13 - 406.1) | 46.69 (11.75 - 142.81) | 0.609 |
| TPOAb | 26.68 (0.84 - 489.9) | 89.66 (15.15 - 267.56) | 0.605 |
| **Radiomics features** |  |  |  |
| RS | 0.15 ± 0.71 | -0.42 ± 0.45 | <0.001 |

PTC: papillary thyroid carcinoma; CLNM: central lymph node metastasis; HT: Hashimoto's thyroiditis; CUS: conventional ultrasound; TT3: total triiodothyronine; FT3: free triiodothyronine; TT4: total thyroxine; FT4: free thyroxine; TSH: thyroid stimulating hormone; TG: thyroid globulin; TGAb: anti-thyroglobulin antibodies; TPOAb: thyroidperoxidase antibodies; RS: radiomics scores

| **Supplemental Table 3. Multivariable analysis of clinical features, CUS features and RS for predicting in PTC patients with HT in the training dataset** | | | |
| --- | --- | --- | --- |
| **Parameter** | **OR** | **95% CI** | **P-value** |
| Age < 41 y | 2.62 | 1.21 - 5.83 | 0.015 |
| Tumor diameter ≥0.9 cm | 2.00 | 0.84 - 4.79 | 0.116 |
| Male | 4.17 | 1.13 - 17.46 | 0.039 |
| Ill-defined margin | 1.23 | 0.54 -2.77 | 0.612 |
| Microcalcification | 1.71 | 0.77 - 3.75 | 0.186 |
| RS | 4.60 | 2.11 - 11.46 | <0.001 |
| OR: odds ratios; CI: confidence intervals | | | |
